# JSRV Intragenic Enhancer Element Increases Expression from a Heterologous Promoter and Promotes High Level AAV-Mediated Transgene Expression in the Lung and Liver of Mice

**DOI:** 10.3390/v12111266

**Published:** 2020-11-06

**Authors:** Darrick L. Yu, Natalie Chow, Sarah K. Wootton

**Affiliations:** Department of Pathobiology, Ontario Veterinary College, University of Guelph, Guelph, ON N1G 2W1, Canada; darrickyu@gmail.com (D.L.Y.); nataliesmchow@gmail.com (N.C.)

**Keywords:** adeno-associated virus (AAV) vector, jaagsiekte sheep retrovirus (JSRV), LTR, enhancer, transduction

## Abstract

Jaagsiekte sheep retrovirus (JSRV) induces tumors in the distal airways of sheep and goats. A putative intragenic enhancer, termed JE, localized to the 3′ end of the JSRV *env* gene, has been previously described. Herein we provide further evidence that the JE functions as a transcriptional enhancer, as it was able to enhance gene expression when placed in either forward or reverse orientation when combined with a heterologous chicken beta actin promoter. We then generated novel composite promoters designed to improve transgene expression from adeno-associated virus (AAV) gene therapy vectors. A hybrid promoter consisting of the shortest JE sequence examined (JE71), the U3 region of the JSRV long terminal repeat (LTR), and the chicken beta actin promoter, demonstrated robust expression in vitro and in vivo, when in the context of AAV vectors. AAV-mediated transgene expression in vivo from the hybrid promoter was marginally lower than that observed for AAV vectors encoding the strong CAG promoter, but greatly reduced in the heart, making this promoter/enhancer combination attractive for non-cardiac applications, particularly respiratory tract or liver directed therapies. Replacement of the murine leukemia virus intron present in the original vector construct with a modified SV40 intron reduced the promoter/enhancer/intron cassette size to 719 bp, leaving an additional ~4 kb of coding capacity when packaged within an AAV vector. Taken together, we have developed a novel, compact promoter that is capable of directing high level transgene expression from AAV vectors in both the liver and lung with diminished transgene expression in the heart.

## 1. Introduction

Adeno-associated virus (AAV) is a small, non-pathogenic, non-enveloped, single stranded DNA virus belonging to the *Parvoviridae* family [1,2]. In 2017, Luxturna (voretigene neparvovec-rzyl), a recombinant vector based on AAV was approved by the U.S. Food and Drug Administration for the treatment of biallelic RPE65 gene mutation-associated retinal dystrophy, a rare form of inherited vision loss that may result [3]. In 2019, a second AAV gene therapy for the treatment of children less than two years of age with spinal muscular atrophy (SMA), termed Zolgensma, was the second directly administered gene therapy approved in the U.S., demonstrating the growing promise of AAV based gene therapies for the treatment of a wide variety of conditions and inherited disorders. 

AAV offers many advantages over other gene delivery vectors such as adenovirus vectors due to its superior transducing efficiency in vivo, its ability to promoter sustained transgene expression, its low immunogenicity and the fact that it can and is being used in a wide range of clinical applications [4]. However, the limited packaging capacity of AAV vectors (~4.7 kb) necessitates the selection of promoter/enhancer elements that are as small as possible, yet retain a high degree of expression, particularly in scenarios where the transgene is of a considerable size, such as in the case of cystic fibrosis transmembrane conductance regulator (CFTR) (4.4 kb). The optimal promoter/enhancer combination for AAV vectored gene therapy applications would be one that has high activity in the target cell population, but minimal to low activity in non-target cells.

Jaagsiekte sheep retrovirus (JSRV) is a simple betaretrovirus that is capable of inducing a form of lung cancer in sheep known as ovine pulmonary adenocarcinoma [5]. Viral gene expression is primarily governed by promoter and enhancer elements located within the long terminal repeat (LTR) sequences that are found on the 5′ and 3′ terminal ends of the integrated provirus [6]. More recently, a putative enhancer sequence, known as JE, has been located outside of the 3′ LTR within the env gene, just prior to the beginning of the 3′ LTR sequence. Adeno-associated virus (AAV) vectors bearing the putative JE enhancer sequence in conjunction with the JSRV long terminal repeat (LTR) demonstrated enhanced, tissue specific expression. Augmentation of the JSRV LTR with the JE resulted in a >4-fold enhancement in lung directed transgene expression and a ~2-fold improvement in liver when vectors were administered to mice [7].

We sought to further delineate the manner in which JE functions and provide further evidence that it is able to function as a transcriptional enhancer. In addition, we hypothesized that promoter/enhancer cassettes based on the JE and the JSRV LTR sequences would be highly effective for in vivo gene delivery purposes, owing to their ability to promote high level protein expression and their relatively compact size.

## 2. Materials and Methods 

### 2.1. Cell Culture

Human embryonic kidney (HEK 293, ATCC CRL-1573) cells, HEK 293T cells, rat fibroblast (208F) cells, and HTX cells, a pseudodiploid subclone of HT-1080 fibrosarcoma cells [8], were maintained in Dulbecco’s modified Eagle’s medium (DMEM; Thermo Fisher Scientific, Ottawa Canada) supplemented with 10% fetal bovine serum (FBS) (GIBCO, Invitrogen), 100 units/mL penicillin, 100 μg/mL streptomycin and 2 mM L-glutamine in a humidified 5% CO_2_ atmosphere at 37 °C.

### 2.2. Molecular Cloning

PCR was used to amplify JE or LTR sequences from the molecular clone of JSRV, pCMV-JS21 [9]. Vectors encoding human alkaline phosphatase reporter gene (hPLAP) were derived from an AAV vector plasmid, AEEE1AP, as described previously [7]. The vector was modified to replace the Enzootic-Nasal Tumor Virus-1 (ENTV-1) enhancer/promoter component with enhancer elements derived from Jaagsiekte Sheep Retrovirus (JSRV), acting on the chicken beta actin promoter to drive expression of hPLAP. Splicing of the hPLAP encoding transcript was promoted by the presence of a murine leukemia virus env intron [10] found between the enhancer/promoter and the hPLAP gene. Following the hPLAP gene was the SV40 polyA tail for the polyadenylation of transcripts. JE and LTR sequences were cloned into XbaI and BglII sites in the AAV vector plasmid, AEEE1AP [7] containing a murine leukemia virus retrovirus intron and hPLAP reporter gene. The chicken beta actin promoter (CBA) was cloned downstream of the JE or JSRV LTR sequences into BglII and KpnI sites. The sequence for the putative enhancers is as follows: JE71: ACATATGAAATATAGAAATATGTTACAGCACCAACATCTTATGGAGCTTTTAAAAAATAAAGAGAGGGGAG; JE184:ACCCTGATTGGTGTAGGAATACTTGTGTTTATTATAATTGTCGTAATCCTTATATTTCCTTGCCTTGTTCGTGGCATGGTTCGCGATTTTCTAAAGATGAGAGTTGAAATGCTGCATATGAAATATAGAAATATGTTACAGCACCAACATCTTATGGAGCTTTTAAAAAATAAAGAGAGGGGAG; and JE324: CGTTAGACCTTTTACAACTGCATAATGAGATTCTTGATATTGAAAATTCGCCGAAGGCTACACTAAATATAGCCGATACTGTTGATAATTTCTTGCAAAATTTATTCTCTAATTTTCCTAGTCTCCATTCGCTGTGGAAAACCCTGATTGGTGTAGGAATACTTGTGTTTATTATAATTGTCGTAATCCTTATATTTCCTTGCCTTGTTCGTGGCATGGTTCGCGATTTTCTAAAGATGAGAGTTGAAATGCTGCATATGAAATATAGAAATATGTTACAGCACCAACATCTTATGGAGCTTTTAAAAAATAAAGAGAGGGGAG. 

### 2.3. Transfection of Mammalian Cells

Approximately 5 × 10^6^ cells were seeded onto three 10-cm tissue culture dishes for each construct 24 h prior to transfection. Cells were transfected with 10 µg each of pCMV-βgal and the construct of interest using polyethylenimine (Polysciences Inc., Warrington, PA, USA) for 208F rat fibroblast cells and calcium phosphate for human HEK 293, HEK 293T and HTX cells. Polyethylenimine transfection was conducted according to manufacturer’s directions. Transfection using the calcium phosphate method was conducted as described previously [11].

### 2.4. Preparation of Cell Lysates

After 48 h, cells were washed with 10 mL of ice cold PBS and scraped off the plate using a rubber policeman into 1 mL of PBS. Cells and PBS were spun down at 4000 rpm for 2 min to recover cells. 500 µL of TMNC lysis buffer (50 mM Tris-HCl (pH 7.5), 5 mM MgCl_2_, 100 mM NaCl, and 4% (wt/vol) 3-[(3-cholamidopropyl)-dimethylammonio]-1-propanesulfonate (CHAPS) was added to each cell pellet before pipetting up and down to suspend the cells. Cells were allowed to lyse for 15 min on ice. Cell debris was removed by centrifuging the sample at 14,000 rpm and the supernatant was recovered for use in subsequent assays. A Bradford assay was performed on cell lysates according to the method of Sambrook and Russell to determine total protein concentration [11].

### 2.5. Beta-Galactosidase Assay

Beta-galactosidase assays were performed by the method of J. Miller [12]. The following solutions were mixed together prior to performing the assay: 100× Mg^2+^ solution containing 0.1 M MgCl_2_ and 4.5 M β-mercaptoethanol, 1× ONPG solution containing 4 mg/mL o-nitrophenyl-β-D-Galactoside (ONPG) dissolved in 0.1 M dibasic sodium phosphate buffer pH (7.5). 3 µL of 100× Mg^2+^, 66 µL of 1× ONPG, 30 µL of cell lysate, and 201 µL of 0.1 M sodium phosphate were mixed together to initiate the reaction. Reactions were incubated at 37 °C until a faint yellow color developed. Reactions were stopped by adding 500 µL of 1 M Na_2_CO_3_. To determine beta-galactosidase activity, absorbance was read at 420 nm using a BioTeK Powerwave XS2 plate reader.

### 2.6. In Vivo Administration of AAV Vectors

Mouse experiments were performed in accordance with the guidelines set forth by the Canadian Council on Animal Care (CCAC). Eight-week old C57BL6/J mice were obtained from Charles River Laboratories (Saint-Constant, QC). AAV vectors were produced by cotransfection of HEK 293 cells with genome and packaging plasmids as described previously [13]. AAV vector titers were determined by quantitative polymerase chain reaction (qPCR) analysis as described elsewhere [14]. AAV vectors were administered via three different routes of administration: intravenous, intraperitoneal, and intranasal to determine relative promoter activity. For intravenous delivery, a phosphate buffered saline (PBS) solution containing 2 × 10^10^ vector genomes of AAV vectors was injected in a 100 µL volume into the tail vein. For intraperitoneal, 8 × 10^10^ vector genomes were injected into the intraperitoneal space in a 500 µL total volume containing the AAV vector plus PBS. For intranasal delivery, 1 × 10^10^ vector genomes were delivered in two aliquots of 40 µL each in order to maximize the chances that tissues deep in the lung would be transduced. A modified method of intranasal delivery was used so as to ensure vector delivery to the distal lung [15]. Mice were euthanized 4 weeks post vector administration, and lungs were perfused through the heart with 20 mL of PBS and then separated into individual lobes. For consistency, the same lung lobe from each mouse was either flash frozen in liquid nitrogen or fixed in 2% paraformaldehyde-PBS for 16 h at 22 °C. Half of other major organs, including the liver, spleen, pancreas, nose, heart, and kidney were fixed for 24 h at 22 °C, with the other half placed into liquid nitrogen for a subsequent enzymatic assay of hPLAP activity. Tissues were stained for vector-encoded heat-stable hPLAP as described previously [7]. Gross pictures of stained tissues were taken using a Zeiss dissecting scope (Zeiss Canada, Toronto, ON, Canada).

### 2.7. Determination of Alkaline Phosphatase Activity

**Figure 1:**
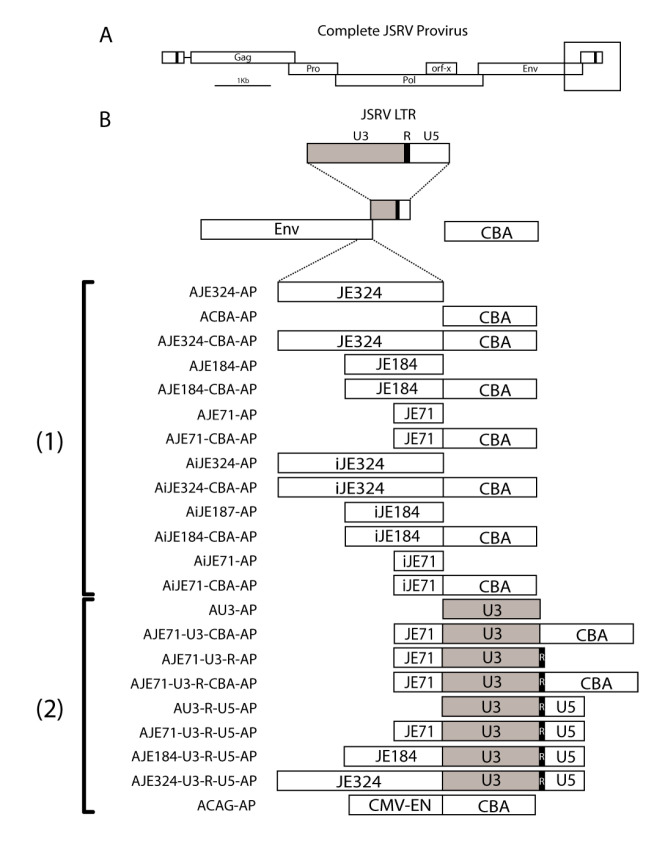
Schematic Diagram of Promoter/Enhancer Constructs. (**A**) The structure of an integrated Jaagsiekte sheep retrovirus (JSRV) provirus. A box is drawn around the 3′ end of the *env* gene and the 3′ long terminal repeat (LTR) where the putative enhancer lies. (**B**) Promoter/enhancer constructs used to drive human placental alkaline phosphatase reporter gene expression and determine if JE functions as a transcriptional enhancer. JE71, JE184, and JE324 refer to three different lengths of the putative JE enhancer region, 71, 184 and 324 bp in length, respectively. The JE sequences are taken from the 3′ end of the *env* gene just prior to the start of the 3′ LTR. In some constructs, the JE was inverted, denoted with an “i”. JE and inverted JE were placed in front of the chicken beta actin (CBA) promoter to determine if they could function upon a heterologous promoter. (2) A number of constructs were also evaluated for their utility as gene expression vectors. Constructs bearing the U3 region of the JSRV LTR, the U3 and R region of the LTR, or the full length JSRV LTR (U3, R and U5 regions), in combination with the chicken beta actin promoter were also tested. A subset of these was evaluated in vivo: ACBA-AP, AJE71-CBA-AP, AJE71-U3-CBA-AP, ACAG-AP (a construct bearing the CAG promoter, which combines the CMV immediate early enhancer, chicken beta actin promoter and rabbit β-globin intron). AP denotes the presence of a human placental alkaline phosphatase reporter gene.

For in vitro studies, cell lysates were heated at 65 °C for 1 h to inactivate endogenous alkaline phosphatases. For 208F cells, 30 µg of total protein as determined by Bradford assay was loaded into each well of a 96 well plate for each 10 cm dish. For HTX, HEK 293, and HEK 293T cells, 100 µg of total protein as determined by Bradford assay were loaded into each well of a 96 well plate. Mouse tissues were harvested 4 weeks after vector administration, snap-frozen in liquid nitrogen and stored at −80 °C until assayed. A small piece (approximately 0.5 cm in diameter; ~30 mg) of the tissue to be analyzed was homogenized in TMNC lysis buffer (50 mM Tris HCl pH 7.5, 5 mM MgCl_2_, 100 mM NaCl, 4% (wt/vol) CHAPS) using a Precellys 24 homogenizer (Bertin Technologies, Montigny-le-Bretonneux, France), with ~200 µL of TMNC buffer in a FastPrep™ Lysing Matrix A tube (MP Bio, Santa Ana, CA, USA). Tissue homogenates were placed in a water bath at 65 °C for 1 h to inactivate endogenous heat-labile AP activity and subsequently clarified by centrifugation at 17,900× *g* for 15 min at 4 °C to remove cell debris. The protein content of each sample was determined by the method of Bradford, and the AP activity in tissue lysates was determined, in triplicate, by a fluorometric assay using the 4-methylumbelliferyl phosphate (MUP) (Sigma, St. Louis, MO, USA) substrate, as described previously [7]. The mean and standard deviation were calculated for each of the different cell lines and vector constructs, as well as for lung, liver, heart, pancreas and spleen for each of the diffesrent vector treatment groups. To correct for differences in transfection efficiency for each of the plasmid constructs described in Figure 1, the mean of AP activity was divided by the mean of β-gal activity. 

## 3. Results

### 3.1. JE on Its Own Functions Poorly as a Promoter

**Figure 2:**
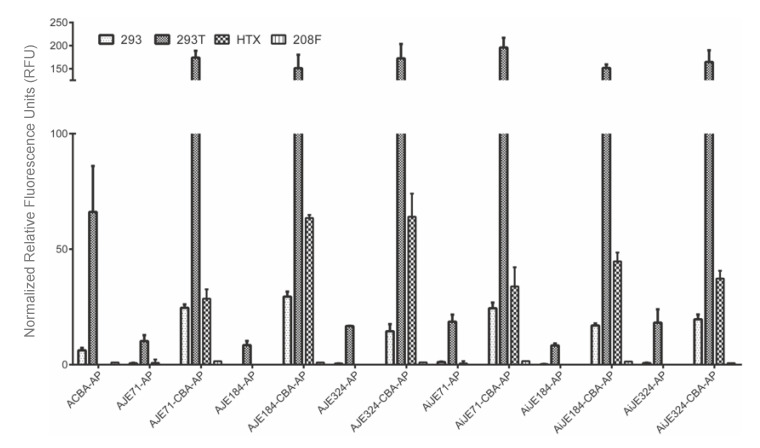
Transfection of four different cell lines (human embryonic kidney (HEK) 293, HEK 293T, rat fibroblast (208F), and human fibrosarcoma (HTX)) with various constructs incorporating the normal orientation JE or the inverted JE. The presence of the inverted JE is denoted by an “i”. Three different sizes were compared corresponding to JE sequences 71, 184, or 324 bp in length. These inverted and non-inverted JE sequences were either placed on their own, or in front of the chicken beta actin promoter to determine if the JE could function as a promoter, or in conjunction with a heterologous promoter (chicken beta actin promoter) as an enhancer. The chicken beta actin promoter was also transfected to indicate a baseline level of expression. Normalized Relative Fluorescence Units (RFU) are reported, where AP activity was normalized to β-galactosidase activity. Experiments were conducted in triplicate with three biological replicates.

Constructs bearing short, medium, or long length JE sequences (pAJE71-AP, pAJE184-AP, and pAJE324-AP, respectively) on their own demonstrated little or no expression when transfected into human embryonic kidney (HEK 293), human fibrosarcoma (HTX), or rat fibroblast (208F) cells (Figure 2). Similarly, constructs bearing inverted versions of short, medium, or long length JE sequences (pAiJE71-AP, pAiJE184-AP, and pAiJE324-AP, respectively) also demonstrated low or no expression in these cell lines. The lack of expression for the JE or inverted JE sequences on their own suggest that the JE is unable to function as a promoter in either forward or reverse orientations in these cell lines. Interestingly, there was a low level of activity in HEK 293T cells, but not 293 cells for forward and reverse JE constructs, suggesting that the SV40 large T antigen was able to promote transcription from the AAV2 inverted terminal repeats (ITR) present on the 5′ flanking end of the JE. No SV40 origin of replication could be found within the plasmid backbone, suggesting that any increase in expression within 293T cells was independent of plasmid replication by the T antigen.

### 3.2. JE Enhances Expression from a Heterologous Promoter, in Both Forward and Reverse Orientations, and Extending JE from the 5’ End Does Not Appear to Further Increase Expression

Inclusion of the chicken beta actin promoter (CBA) markedly increased expression from constructs bearing the JE in all cell lines tested (Figure 2). Comparing pAJE71-AP (possessing the short JE, but lacking the chicken beta actin promoter) to pAJE71-CBA-AP (possessing both short JE and the chicken beta actin promoter), a nearly 20-fold increase was observed in 293T cells and an equivalent or greater increase was observed in 293 and HTX cell lines. In a similar manner, comparing pAJE184-AP (possessing medium length JE alone) to pAJE184-CBA-AP (possessing medium length JE plus the chicken beta actin promoter) and pAJE324-AP (possessing the long version of JE alone) to pAJE324-CBA-AP (long JE in addition to the chicken beta actin promoter), there was a ~20-fold increase in expression in 293T cells and a further increase in other cell lines. Extension of the JE from 71 to 184 and 324 base pairs did not appear to confer a corresponding increase in expression, suggesting that the putative enhancer element is located within the original 71 bp region and no additional enhancer elements are located within the 184 or 324 bp regions. A 2-fold or greater increase in transgene expression was also observed when comparing short, medium, or long JE sequences in conjunction with the chicken beta actin promoter relative to the chicken beta actin promoter on its own, demonstrating that it is not merely the chicken beta actin promoter that was able to confer higher expression.

For another series of constructs, the putative JE sequences were placed in an inverted orientation relative to their orientation in the JSRV provirus. Inverted JE sequences corresponding to short, medium and long lengths placed in front of the chicken beta actin promoter (pAiJE72-CBA-AP, pAIJE184-CBA-AP, and pAiJE324-CBA-AP, respectively) all demonstrated a similar improvement as their non-inverted counterparts in 293, HTX, 293T and 208F cells, while constructs containing the inverted JE (pAiJE72-AP, pAiJE184-AP, and pAiJE324-AP) of varying lengths but lacking the chicken beta actin promoter conferred low expression in all cell lines but 293T. 

### 3.3. A Hybrid Promoter Consisting of JE, the U3 and R Regions of the LTR, and the Chicken Beta Actin Promoter Is Highly Active in a Variety of Cell Lines

**Figure 3:**
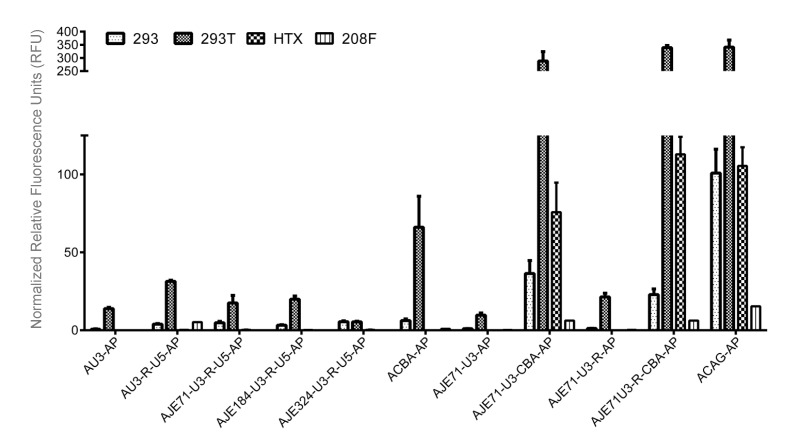
Transfection of four different cell lines (HEK 293, HEK 293T, 208F, and HTX) with various constructs incorporating JE, the chicken beta actin promoter (CBA), and components of the JSRV LTR to determine their suitability for in vivo gene delivery experiments. The JE was investigated to see if it would work with its own homologous promoter, the JSRV LTR, consisting of U3-R-U5 regions, or in conjunction with just the U3/U3 and R regions to enhance expression from the chicken beta actin promoter. These hybrid promoter/enhancer cassettes were compared to the strong CAG promoter and the CBA promoter on its own. Normalized Relative Fluorescence Units (RFU) are reported, where AP activity was normalized to β-galactosidase activity. Experiments were conducted in triplicate with three biological replicates.

The relatively low activity of the wild-type JSRV promoter is dwarfed by the high activity brought about by the combination of the JE, the U3 and R regions of the JSRV LTR placed upstream of the chicken beta actin promoter (pAJE71-U3R-CBA-AP) in 293, 293T, 208F, and HTX cells. In fact, expression was so high in 293T and HTX cells that it matched or exceeded expression by the CAG promoter (pACAGAP), currently one of the best promoters for constitutive expression from a variety of cell lines (Figure 3). A vector consisting of the JE71, U3, but not the R region of the JSRV LTR (pAJE71-U3-CBA-AP) expressed to high levels in the same cell lines, reaching levels comparable to the CAG promoter (Figure 3). Both pAJE71-U3-CBA-AP and pAJE71-U3R-CBA-AP demonstrate expression that was much higher than a vector encoding a promoter consisting of the CBA promoter alone, demonstrating the ability of the JE and JSRV LTR elements to enhance expression from a heterologous promoter.

Constructs possessing components of the JE or JSRV LTR but lacking the chicken beta actin promoter demonstrated low activity in 293, 293T, 208F and HTX cell lines (Figure 3). pAU3-AP, pAU3-R-U5-AP, pAJE72-U3-R-U5-AP, pAJE184-U3-R-U5-AP, pAJE324-U3-R-U5-AP, pAJE71-U3-AP, and pAJE71-U3-R-AP each exhibited poor expression in 293, 293T, 208F and HTX cells. This may be due to the high specificity of JSRV LTR components for tissues of the respiratory tract, the primary target for JSRV infection.

### 3.4. The JE/JSRV LTR/CBA Hybrid Promoter Is Active in the Lungs, Nose, Trachea, Liver, Heart, Spleen, and Pancreas of Mice

**Figure 4:**
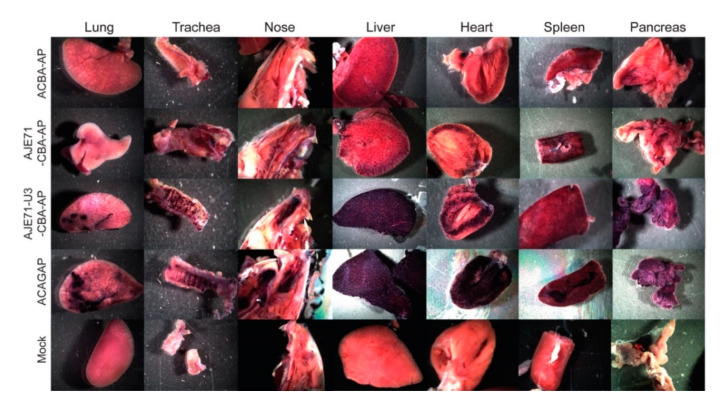
Representative gross images of tissues stained to indicate the presence of human placental alkaline phosphatase reporter gene expression from mice transduced with various JE/CBA constructs. AAV vectors were administered to mice by three different routes of administration concurrently: intravenous (2 × 10^10^ vg), intraperitoneal (8 × 10^10^ vg), and intranasal (1 × 10^10^ vg) to determine relative promoter activity. Mice were euthanized 4 weeks post vector administration and tissues stained for human alkaline phosphatase reporter gene (hPLAP) expression.

**Figure 5:**
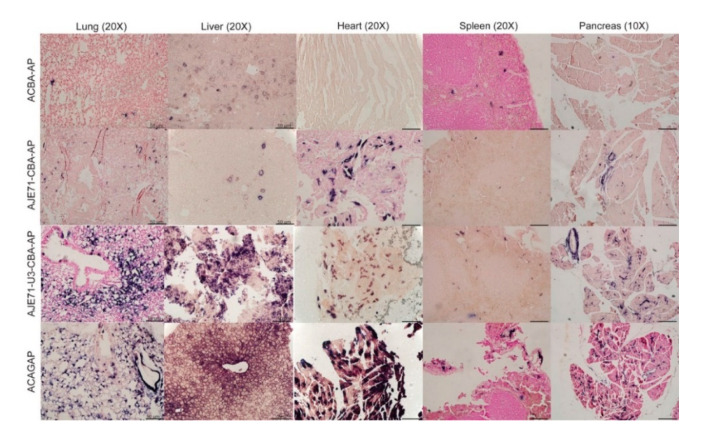
Representative histological alkaline phosphatase staining of tissue sections from mice transduced simultaneously via the intravenous (2 × 10^10^ vg), intraperitoneal (8 × 10^10^ vg), and intranasal (1 × 10^10^ vg) routes with various AAV vectors bearing JE/CBA promoter/enhancer sequences and imaged 4 weeks post-transduction. Scale bar represents 50 µM.

Expression of the JSRV promoter is primarily limited to the respiratory tract (lung, nose, trachea) and liver when mice are transduced via an AAV vector [8]. Inclusion of the JE enhancer element appeared to improve expression in these tissues, as well as enhance expression in the spleen and pancreas in mice transduced with AAV vectors via the intranasal, intraperitoneal and intravenous route (Figure 4). A hybrid promoter consisting of the JE71, U3 region of the JSRV LTR, and CBA promoter (AJE71-U3-CBA-AP) was highly active in a variety of tissues when transduced into mice via AAV vectors. High levels of expression could be seen in the tissues of the respiratory tract, including the lung, nose and trachea, as well as the liver, spleen and pancreas both grossly (Figure 4) and histologically (Figure 5). Unlike the JSRV LTR, expression was also observed in the heart, but not to the same extent as the CAG promoter construct, which demonstrated an extremely high level of expression (Figure 4). Note that since the vectors were all packaged into the same AAV capsid, in this case AAV serotype 6 (AAV6), the differences in reporter gene expression observed in vivo are due to the tissue specificity of the promoter and not the capsid. Moreover, the use of a heat stable placental alkaline phosphatase reporter gene (hPLAP) allows for the heat inactivation of all endogenous alkaline phosphatase while retaining hPLAP enzymatic activity. As such, alkaline phosphatase staining, as evidence by the purple color, is only observed if the tissue has been transduced by the AAV vector expressing hPLAP. This can be observed in the mock infected tissues, where no purple staining was detected, either grossly or histologically. 

In histological sections, strong AP expression could be observed in lung alveolar cells and liver hepatocytes of mice transduced with a vector that bore the short JE and the CBA (AJE71-CBA-AP), similar to that of ACAGAP (Figure 5). Histological sections of heart transduced with ACAGAP demonstrate very dark staining of individual cells transduced with this vector, compared to the AJE71-U3-CBA-AP, which exhibits a fainter degree of staining (Figure 5). Efficient expression was also observed in pancreatic cells for AJE71-U3-CBA-AP and ACAGAP. Within the spleen, it appeared that no vector was particularly effective.

**Figure 6:**
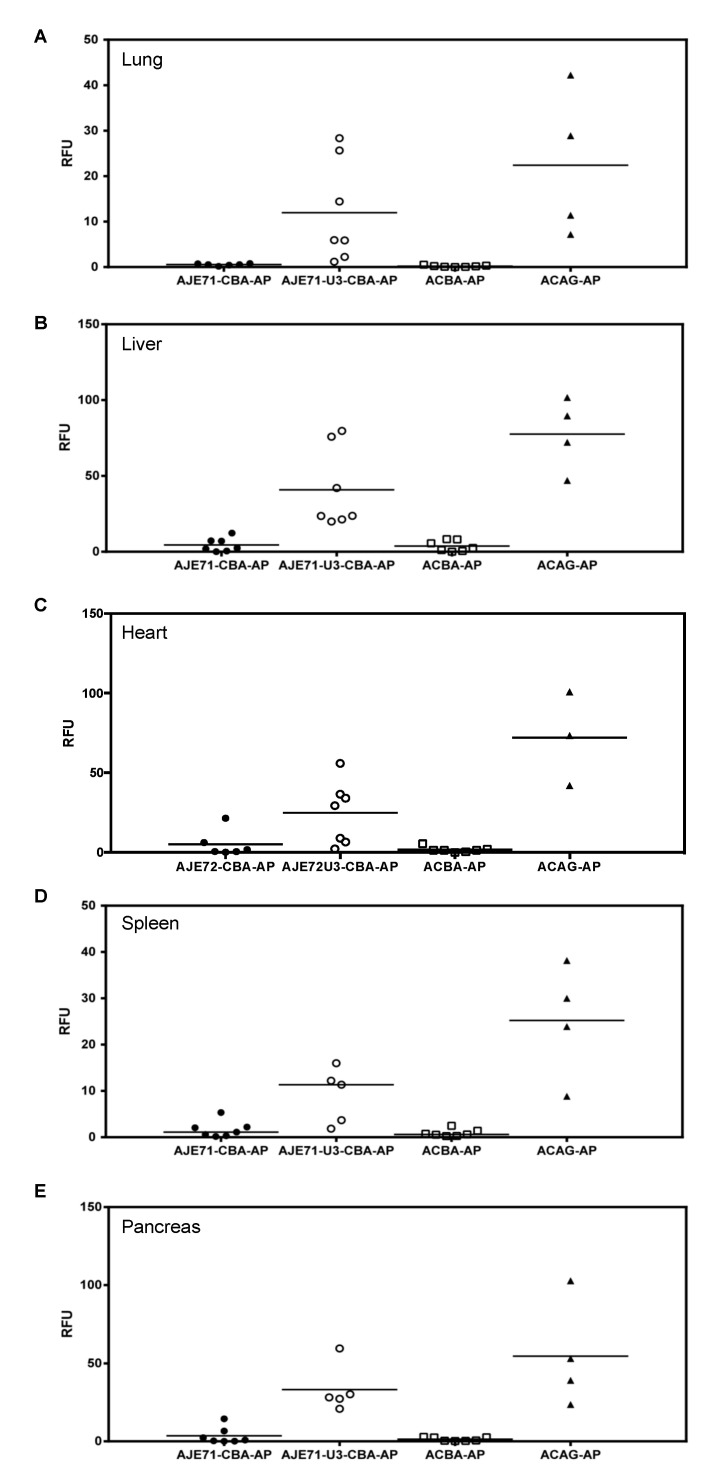
Quantification of alkaline phosphatase activity within homogenized (**A**) lung, (**B**) liver, (**C**) heart, (**D**) spleen and (**E**) pancreas from mice transduced via the intravenous (2 × 10^10^ vg), intraperitoneal (8 × 10^10^ vg), and intranasal (1 × 10^10^ vg) routes with various AAV vectors and harvested for hPLAP enzyme activity analysis 4 weeks post-transduction.

Quantification of hPLAP enzymatic activity in tissues harvested from the vector transduced mice revealed that in all tissues evaluated, expression from AJE71-U3-CBA-AP was reduced relative to ACAG-AP, but was much higher than ACBA-AP. AJE71-CBA-AP was marginally higher than ACBA-AP but far lower than AJE71-U3-CBA-AP, demonstrating the utility of the JSRV U3 region (Figure 6).

### 3.5. A Shorter Intron Can Be Used in Conjunction with AJE71-U3-CBA-AP without Reducing Transgene Expression

**Figure 7:**
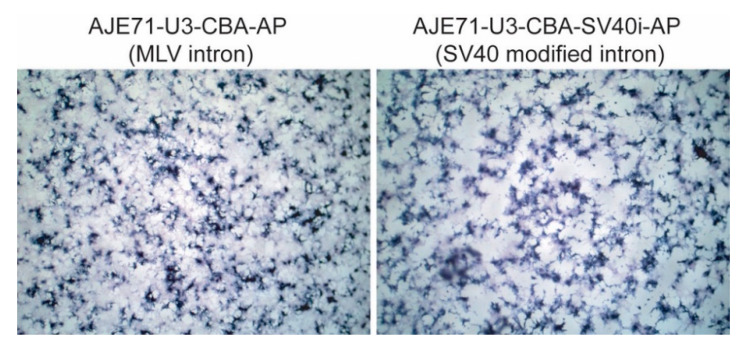
Transfection of two different plasmids in HEK 293 cells, utilizing different introns, employing the JE71-U3-CBA promoter. In the image on the left, an intron derived from MLV is used. In the image on the right, a modified SV40 intron was utilized, reducing the size of the promoter–enhancer–intron combination by 488 bp while maintaining a similar amount of expression. Experiments were conducted in triplicate with three biological replicates. Images were taken at 100× magnification.

The MLV env intron (581 bp) was exchanged for a shorter sequence based on an optimized SV40 intron (93 bp) modified to include consensus splice sites. Transfection of HEK 293 cells with the SV40 intron containing construct compared to the parental plasmid containing the MLV intron demonstrated no visible difference in hPLAP expression (Figure 7).

## 4. Discussion

In its native context, within an integrated JSRV provirus, JE may function to enhance expression from the 5’ LTR sequence to drive viral gene expression, or may participate in the process of tumor development by dysregulating the expression of cellular proto-oncogenes.

A lack of expression was observed in constructs bearing only the JE, compared to constructs that possessed the JE and a functional heterologous promoter, the chicken beta actin promoter. Furthermore, inclusion of the JE in any of its varying lengths described here and in either forward or reverse orientations proved to show an increase in protein expression when combined with the chicken beta actin promoter. These characteristics strongly suggest that the JE is able to function as an enhancer sequence [16]. Extension of the JE sequence did not appear to further enhance expression in the cell lines tested, suggesting that no additional enhancer sequences are localized to the area immediately upstream of the 3′ LTR, or at least ones that are active in the cell lines tested. In addition, lengthening of the JE increased the distance between the promoter and inverted terminal repeat of the AAV vector, and no further increase in expression was observed, suggesting that the distance of the promoter from the ITR was not a factor in increasing expression, as previously hypothesized [7]. The particular region where the JE exists overlaps with an RNA export element termed the SPRE or RejRE, which functions in conjunction with a region overlapping the signal peptide of the JSRV envelope to facilitate export of unspliced genomic RNA [17]. However, it is unlikely that the JE functions as an RNA export element in this context as the JSRV envelope is not present in any of the experiments described. Furthermore, there should be no deficit in the ability of transcripts to be exported from the nucleus as they all contain well characterized introns: either the rabbit β-globin intron for the ACAGAP construct or the Moloney murine leukemia virus intron for the other constructs assayed. In addition, the JE sequence should not be transcribed at a high level as it preceded the sequence encoding the promoter in all of the constructs where it was present. Previous attempts at 5′ Rapid Amplification of cDNA Ends (RACE) and RT-PCR were not able to detect the presence of any transcripts except those originating from the R region, when JE71 was placed in front of the JSRV LTR [7]. 

Low levels of expression observed for the constructs containing JE alone, in either forward or inverted orientation (pAJE71-AP, pAJE184-AP, pAJE324-AP, pAiJE71-AP, pAiJE184AP, and pAiJE324-AP) indicate that JE itself is not able to function effectively as a promoter. This rules out the possibility of JE functioning as a promoter for a transactivating non coding RNA, as is the case in Moloney murine leukemia virus and feline leukemia virus, which both express noncoding RNAs from their 3′ LTRs that are able to transactivate signaling pathways involved in cancer [18,19,20].

Evidence for a possible expression enhancing region or putative enhancer sequence located in regions flanking the JSRV env gene came to light in a paper by Sinn et al., wherein greatly increased JSRV envelope pseudotyped lentiviral vector titers were observed when env flanking sequences were included in the envelope expression cassette [21]. Previous work with the Prague and Schmidt-Ruppin strains of Rous sarcoma virus has demonstrated that sequences immediately preceding the 3′ LTR are able to enhance expression in reporter gene studies [22,23]. Furthermore, precedence exists for the presence of a region upstream of the 3′ LTR determining the spectrum of disease observed in other retroviruses, such as the exogenous virus-specific region (XSR) sequence of the Prague strain of Rous sarcoma virus, which was demonstrated to be a determinant of oncogenicity [24] and a similar region known as the E region in avian leukosis virus (subgroup J) that has been shown to contribute to oncogenicity in certain chicken breeds [25].

**Figure 8:**
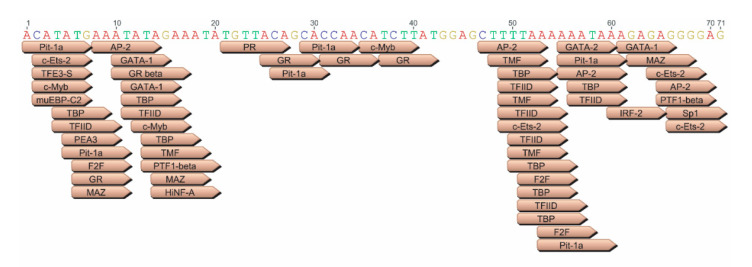
Transcription Factors Predicted to Bind to JE-71. A number of different transcription factors have been predicted to bind to the JE-71 sequence using the EMBOSS 6.5.7 tool tfscan and graphically presented by Geneious. Particularly promising putative transcription factors with multiple predicted binding sites include AP-2 (4 sites), glucocorticoid receptor (GR/GR beta, 5 sites), pituitary-specific positive transcription factor 1 (Pit-1A, 5 sites), and c-ETS-2 (4 sites).

The identity of the transcription factor that binds to the JE has yet to be elucidated; however, one promising candidate is AP-2, for which there are four predicted sites within the JE71 sequence (Figure 8). A wide variety of other transcription factors are also predicted to bind; these include glucocorticoid receptor (GR), for which there are five sites, pituitary-specific positive transcription factor 1 (Pit-1A), for which there are five sites, and c-ETS-2, for which there are four predicted sites. Activation of these pathways through overexpression of transcription factors and/or using pharmacological agents could help to identify which one is responsible for the observed enhancer activity.

A high level of expression could be observed for AJE71-U3-CBA-AP in vivo, nearly matching ACAGAP for some individual mice (Figure 4, Figure 5 and Figure 6). However, on an aggregate level, expression was generally lower for all of the organs assayed. Amongst the biggest difference was in the heart, where expression was >2-fold higher in ACAGAP compared to AJE71-U3-CBA-AP. This property might make the JE71-U3-CBA promoter particularly useful when widespread, constitutive expression in a variety of organs is called for, but expression in the heart is to be minimized. The high level of expression observed in the respiratory tract and liver may make this promoter effective for respiratory tract diseases such as lung, paranasal sinus, and nasal cavity cancer, or a monogenic disorder such as cystic fibrosis. At approximately 650 bp in size, JE71-U3-CBA is not particularly large for a promoter, and may be combined with a short intron such as an optimized SV40 intron (93 bp) for a total enhancer–promoter–intron size of approximately 743 bp, leaving ~4 kb for the transgene and polyA signal when used in the context of an AAV vector, which has a coding capacity of ~4.7 kb. This might be attractive for the design of an AAV vector based therapeutic employing the truncated CFTR fragment previously shown to be able to rescue the processing of endogenous FΔ508 CFTR in vivo [25]. JE71-U3-CBA and JE71-U3R-CBA enhancer/promoter combinations functioned as effectively as the CAG promoter in the cell lines tested, highlighting the potential of these sequences as alternatives to the commonly used CAG promoter (1621 bp) for in vitro protein expression purposes in mammalian cells.

In summary, we demonstrate the presence of an intragenic transcriptional enhancer element, found within the 3′ end of the env gene. This JSRV-based enhancer element demonstrated strong expression in a variety of tissues, particularly respiratory and hepatic, and promoter/enhancer/intron cassettes derived from these elements can be pared down to a sufficiently small size (<630 bp in length), suitable for genome constraints imposed by AAV vector systems.
